# Exosomes repairment for sciatic nerve injury: a cell-free therapy

**DOI:** 10.1186/s13287-024-03837-7

**Published:** 2024-07-18

**Authors:** Guang-Da Xin, Xue-Yan Liu, Xiao-Di Fan, Guan-Jie Zhao

**Affiliations:** 1 Nephrology Department, China-Japan Union Hospital of Jilin University, Changchun, Jilin Province 130000 China; 2 Cardiology Department, China-Japan Union Hospital of Jilin Universit, Changchun, Jilin Province 130000 China; 3 Department of Anesthesiology, China-Japan Union Hospital of Jilin University, Changchun, Jilin Province 130000 China

**Keywords:** Sciatic nerve injury, Exosome, Peripheral nervous, Nerve

## Abstract

Sciatic nerve injury (SNI) is a common type of peripheral nerve injury typically resulting from trauma, such as contusion, sharp force injuries, drug injections, pelvic fractures, or hip dislocations. It leads to both sensory and motor dysfunctions, characterized by pain, numbness, loss of sensation, muscle atrophy, reduced muscle tone, and limb paralysis. These symptoms can significantly diminish a patient’s quality of life. Following SNI, Wallerian degeneration occurs, which activates various signaling pathways, inflammatory factors, and epigenetic regulators. Despite the availability of several surgical and nonsurgical treatments, their effectiveness remains suboptimal. Exosomes are extracellular vesicles with diameters ranging from 30 to 150 nm, originating from the endoplasmic reticulum. They play a crucial role in facilitating intercellular communication and have emerged as highly promising vehicles for drug delivery. Increasing evidence supports the significant potential of exosomes in repairing SNI. This review delves into the pathological progression of SNI, techniques for generating exosomes, the molecular mechanisms behind SNI recovery with exosomes, the effectiveness of combining exosomes with other approaches for SNI repair, and the changes and future outlook for utilizing exosomes in SNI recovery.

## Introduction

SNI, a common peripheral nerve injury typically caused by trauma such as contusions, sharp force injuries, drug injections, pelvic fractures, or hip dislocations, leads to sensory and motor dysfunctions including pain, numbness, loss of sensation, muscle atrophy, reduced muscle tone, and limb paralysis, significantly affecting the patient’s quality of life [1–4]. Wallerian degeneration follows SNI, triggering a variety of signaling pathways, inflammatory factors, and epigenetic regulators [5–7]. To date, numerous approaches have been explored to treat SNI in clinical practice, including nerve tissue and stem cell transplantation, the administration of neurotrophic drugs and nerve growth factors, as well as physical and laser therapy [8, 9]. Direct nerve anastomosis is only suitable for cases involving short gaps [10]. Autologous nerve transplantation is a viable option for addressing large nerve defects, but the effectiveness of this approach, particularly when implemented using microsurgical techniques, is hindered by several challenges, including insufficient availability of nerves sources, potential donor site dysfunction and discrepancies of donor nerve and graft size [11, 12]. Although various surgical and non-surgical therapies have been approved for clinical use, their therapeutic effects remain unsatisfactory. Therefore, there exists a pressing necessity to explore novel therapeutic interventions for SNI [13].

Exosomes are endoplasm-derived extracellular vesicles, ranging from 30 to 150 nm in diameter, that encapsulate a diverse array of bioactive molecules, predominantly nucleic acids and proteins, which play an indispensable role in facilitating intercellular communication [14–17]. Recent studies have revealed that exosomes possess favorable characteristics such as good stability, biocompatibility, the ability to permeate biological barriers, low immunogenicity, therapeutic targeting, and prolonged blood circulation [18–20]. Genetically engineered cells can endow exosomes with new functions by equipping them with functional proteins, enhancing their capability for specific therapeutic and diagnostic applications [21, 22]. Moreover, exosomes can serve as transporters carrying small molecules or nucleic acid medications for the targeted delivery of drugs to specific cells or tissues [23–25]. Fayazi et al. suggests that exosomes derived from stem cells may exhibit neuroprotective capabilities in SNI by facilitating myelin regeneration and functional recovery [26]. Researchers have found that exosomes from various stem cell sources can promote the repair of damaged neural tissue [27, 28]. Exosomes are known to play a crucial role in the repair of SNI, yet there is a scarcity of research elucidating the specific mechanisms by which exosomes facilitate this repair process. Herein, this review delves into the pathological progression of SNI, techniques for generating exosomes, the molecular mechanisms behind SNI recovery with exosomes, the effectiveness of combining exosomes with other approaches for SNI repair, and the changes and future outlook for utilizing exosomes in SNI recovery.

## Pathological mechanism of SNI

The pathological process following SNI primarily involves changes in the phenotype of Schwann cells (SCs) and the activation of macrophages [29]. Eliminating residual myelin is a crucial step in restoring the sciatic nerve after demyelination because residual myelin can have an inhibitory effect [30]. Various genes or proteins regulate and facilitate the phagocytosis of myelin fragments by SCs and macrophages [31–33]. Once myelin fragments are cleared, SCs migrate and cluster together to form myelin, which protect the axon and facilitate its repair and regeneration [34].

### Phenotypic changes in SCs

**Fig. 1:**
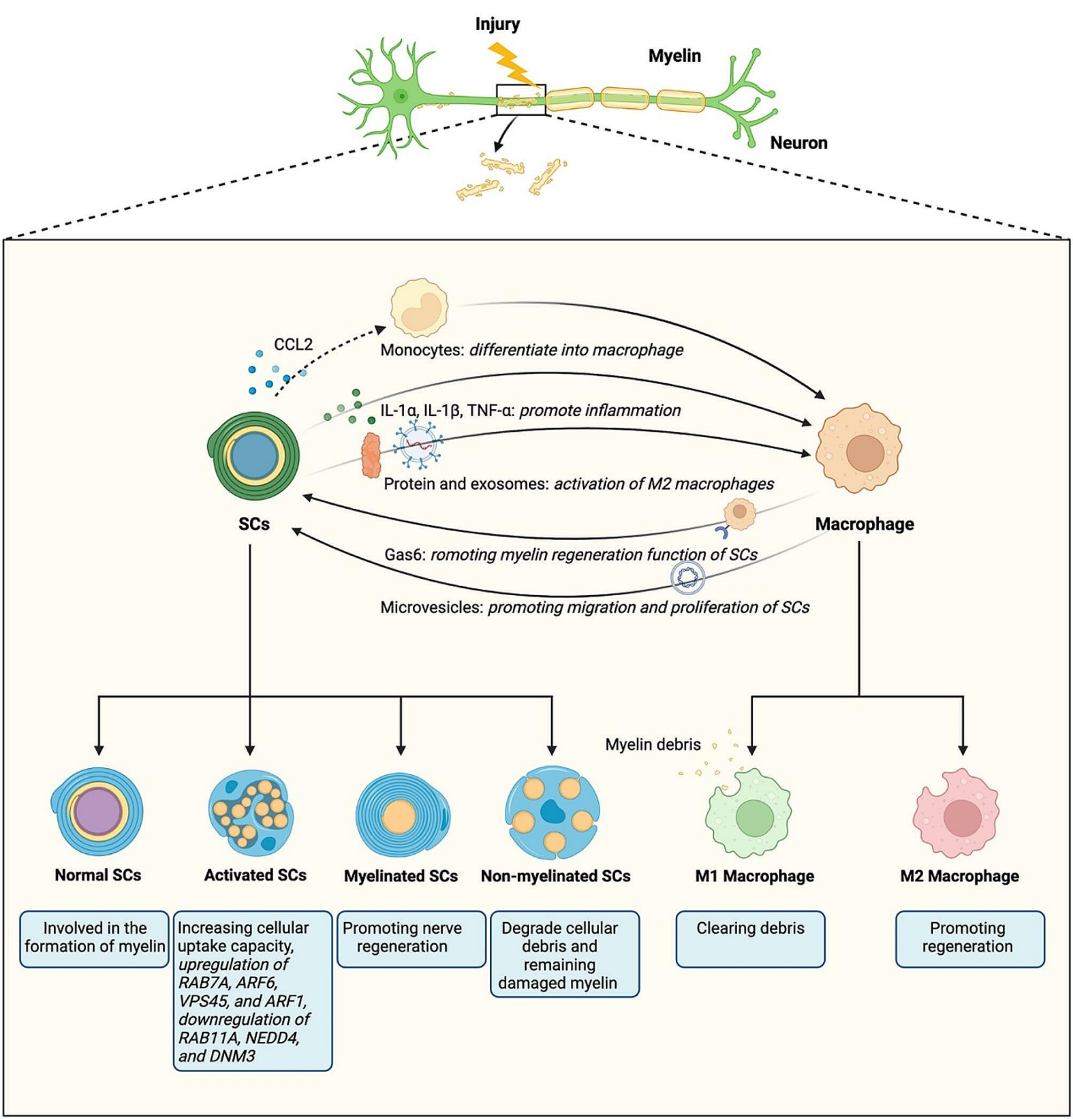
Pathological mechanisms of SNI. SCs, Schwann cells; CCL2, C C motif ligand 2; IL-1α, Interleukin-1α; IL-1β, Interleukin-1β; TNF-α, Tumor Necrosis Factor-α

SCs are a type of glial cells responsible for forming and maintaining the myelin around axons, as well as promoting axon regeneration. The peripheral nervous system (PNS) possesses an inherent regenerative ability primarily attributed to SCs [35, 36]. Researchers have demonstrated that activated SCs exhibit increased cellular uptake capacity compared to normal SCs, potentially due to changes in specific proteins such as the upregulation of RAB7A, ARF6, VPS45, and ARF1, and the downregulation of RAB11A, NEDD4, and DNM3 [37]. These findings provide new insights into the mechanisms of myelin fragment clearance and myelin restoration during the subacute phase of nerve damage. Moreover, SCs can be divided into non-myelinated and myelinated phenotypes. Myelinated SCs are the dominant glial cell type in the PNS and play a crucial role in various aspects, including the development, maintenance, regeneration, and overall functioning of peripheral nerves [38]. Non-myelinated SCs, known as Remak cells, provide support to fine caliber axons during both normal conditions and after injury, while the myelinated SCs in the distal nerve stump become dedifferentiated and activated following SNI [39]. In the process recognized as Waller degeneration, the SCs dedifferentiated into non-myelinated type, and then begin to degrade cellular debris and remaining damaged myelin [40]. Additionally, the exchange of interaction and information between dedifferentiated SCs and other cells is crucial for regeneration after a SNI. This communication involves various bioactive components, including lipids, proteins, mRNA, lncRNA and circRNA [41–43] (Fig. 1).

### Activation of macrophages

Macrophages exhibit significant variability in their behavior and can differentiate into distinct phenotypes based on cues from their surrounding environment. They can polarize into either M1 macrophage, a pro-inflammatory phenotype, which is identified by the presence of CD86 markers on their surface, or M2 macrophage, an anti-inflammatory phenotype, marked by CD206. This polarization allows macrophages to adapt and respond appropriately to different physiological conditions, playing versatile roles in the immune system’s response to various stimuli or injuries [44, 45]. The neuroinflammatory response triggers peripheral immune cells, such as circulating macrophages, to gather or accumulate at the site of injury [46].

After an injury, macrophages are key immune cells that migrate towards the site of the damage. Their role is multifaceted. Initially, they help clear the debris, such as phagocytizing myelin remnants, which is essential because these remnants can inhibit regeneration if not removed [47]. By clearing these inhibitory materials, macrophages prevent them from suppressing the signals that encourage regrowth and repair [48]. Additionally, macrophages switch roles from being primarily phagocytic to promoting regeneration. They release factors that stimulate the regrowth of axons, the long nerve fibers that are often damaged during injuries [49]. This switch is crucial for the transition from an inflammatory state, which is necessary for cleaning up the site of injury, to a regenerative state, which is essential for healing and the restoration of function [47–49]. Thus, macrophages are indispensable not only for their role in debris clearance but also in facilitating and promoting the healing and regrowth of nerve tissues post-injury.

### Interaction between SCs and macrophages

SCs play a pivotal role in the recruitment of macrophages to the site of nerve injury through the secretion of various ligands, including cytokines and chemokines. These signaling molecules are crucial for initiating and guiding the immune response following nerve damage. Key cytokines and chemokines released by SCs include: firstly, C C motif ligand 2 (CCL2): This chemokine is particularly important for attracting monocytes, which can differentiate into macrophages, to the injury site. Secondly, IL-1α and IL-1β: these interleukins are pro-inflammatory cytokines that help in amplifying the inflammatory response, which is essential for clearing the damaged tissue and preparing for regeneration. Thirdly, TNF-α: tumor necrosis factor-alpha is another pro-inflammatory cytokine that promotes inflammation and aids in the recruitment and activation of macrophages. By secreting these molecules, SCs actively contribute to the inflammatory environment needed for healing and regeneration, signaling macrophages to come to the injury site where they can perform their crucial roles in debris clearance and tissue repair [50–53]. Fourth, SCs-derived gel protein [54] and periosteal protein [55] are also important in promoting macrophage motility and infiltration into the injury site in vivo. Fifth, in Schwan cell-derived exosomes (SCs-Exo), a lncRNA called axon regeneration-associated transcription is upregulated after SNI to enhance axon regeneration and promote recovery of motor function through recruitment and activation of pro-regenerative macrophages [56]. Meanwhile, macrophages also play a crucial role in regulating the maturation process of SCs after nerve injury. Macrophages express a protein called Growth arrest-specific 6 (Gas6) that is involved in controlling the function of SCs. The absence of Gas6 within macrophages leads to an altered response from SCs and impairs their ability to effectively remyelinate [57]. Moreover, microvesicles released by CD206 + macrophages aid in promoting migration and proliferation of SCs [58]. Therefore, this interaction between macrophages and SCs is essential for promoting proper nerve regeneration.

## Preparation and functions of exosomes

### Preparation

In the realm of exosomes research and their practical application, the identification and extraction techniques of exosomes form the basis of the study. Although it presents a challenge to achieve complete isolation of exosomes from other types of extracellular vesicles, a number of standardized methods for the identification and extraction of exosomes have been established and are used by researchers. Various techniques can be employed to isolate exosomes from cell cultures or body fluids, depending on their size and source. To date, researchers have developed five types of exosome separation techniques: Ultra-high-speed centrifugation, size-based separation technology, in situ polymer precipitation, immunoaffinity capture technique and, more recently, microfluidic derived technology [59]. The most common separation methods used in exosome studies are ultra-high speed centrifugation and exosome extraction kits based on polyethylene glycol precipitation [60]. Various techniques have been utilized in the pursuit of comprehending the constitution of exosomes. These methods encompass transport electron microscopy, scanning electron microscopy, cryo-electron microscope, atomic force microscopy, nanoparticle tracking analysis, dynamic light scattering, trypsin digestion, mass spectrometry, enzyme linked immunosorbent assay analysis, labeled protein expression detection such as Western blot analysis and flow cytometry and these approaches rely on the scrutiny of exosome morphology, particle size, and surface markers for the purpose of exosome identification [61–63].

### Functions

Researchers are constantly collecting and analyzing exosomes from various biopsies to discover the dynamic properties and molecular features of exosomes and to exploit the powerful functions of exosomes. In general, exosomes have diagnostic, therapeutic and prognostic effects. The inherent architecture and distinctive cellular functionalities of exosomes render them exceptionally promising as conveyors of natural medication or genetic material, possessing substantial diagnostic capabilities within the realms of regenerative medicine, immunotherapy, and vaccination experiments. However, to maximize their potential, it is imperative to establish dependable and effective techniques for isolating these vesicles [64]. Exosomes exhibit significant potential as diagnostic biomarkers for a range of medical conditions, including cardiovascular diseases, Parkinson’s disease, other neurodegenerative diseases such as Alzheimer’s disease and cancer [65]. Treatment strategies for exosomes can be classified into three categories, namely direct interventions, indirect interventions, and alternative therapies. Direct therapy takes advantage of exosomes’ ability to transfer proteins and nucleic acids between cells, using exosomes as carriers of drugs to treat diseases or injuries [65]. Exosomes serve as biomarkers when employing indirect techniques. Alternative strategies seek to impede the advancement of diseases by interrupting the circulation of exosomes that also include harmful disease-associated cargo such as viral miRNA and proteins [66], immunosuppressive factors promoting tumor metastasis [67] or tumor signaling molecules impeding therapeutic medications [68]. An increasing body of research has demonstrated that exosomes play a critical role in the process of tumorigenesis, growth, metastasis and drug resistance [69]. The loading of exosomes originating from tumors aligns with the genetic composition of parental tumor cells [70]. Furthermore, exosomes exhibit remarkable stability in circulation, effectively safeguarding their contents against degradation [71]. Therefore, exosomes and their transport products are beginning to be considered as novel biomarkers for cancer prognosis assessment.

## Exosomes for the treatment of SNI

### Stem cell-derived exosomes repair SNI

#### ADSCs-Exo

Advancing research into the mechanisms of sciatic nerve regeneration, identifying novel therapeutic targets, and exploring innovative treatment methods are crucial steps toward enhancing the therapy for SNI. Recent research has shown that adipose-derived stem cells (ADSCs), when cultured with SCs, can enhance myelin-related markers, playing a significant role in nerve injury repair and regeneration [72]. In their in vitro studies, Wang and colleagues discovered that a hypocapnic environment stimulates adipose-derived stem cells (ADSCs) to release smaller exosomes, which more efficiently concentrate and transport intracellular miR-218 to recipient cells. Subsequent in vivo studies demonstrated that this process further accelerates SNI healing by enhancing myelination, promoting tissue regeneration, and restoring motor function [73]. Chen and colleagues evaluated the efficacy of ADSCs-Exo in a rat model of sciatic nerve transection with a 10 mm gap. Compared to the control group, the ADSCs-Exo treatment group exhibited significant improvements in axon regeneration, myelination, and recovery from denervated muscle atrophy [74]. FK506, an immunosuppressant known to promote nerve regeneration, was studied for its effects when combined with ADSCs-Exo after treatment. In a murine model of sciatic nerve compression injury, the local application of these ADSCs-Exo significantly reduced macrophage autophagy following spinal segmental nerve compression injury [75]. Proteomic analysis of ADSCs-Exo revealed that HSPA8 and EEF1A1 show promising potential in reducing autophagy through exosome mediation [75]. Additionally, HDAC, ITGB1, and APP have been identified as potential candidates involved in the exosome-mediated enhancement of nerve regeneration [76]. Furthermore, Yin and colleagues administered ADSCs-Exo to rats with SNI via tail vein injection. Their results indicate that ADSCs-Exo positively influence neuronal axon regeneration by reducing SCs apoptosis, decreasing autophagy, and encouraging Bungner ligament formation, thereby promoting fiber regeneration following SNI [77]. They also found that ADSCs-Exo moderately reduced autophagy in damaged SCs by downregulating Kpna2 through miRNA-26b, facilitating myelin regeneration [78]. ADSCs-Exo can promote SNI regeneration through up-regulating the expression of Bcl-2 mRNA and down-regulating the expression of Bax mRNA, thereby reducing cell apoptosis and promoting the proliferation of SCs [42].

#### Mesenchymal stem cell-derived exosomes (MSCs-Exo)

Mesenchymal stem cells (MSCs) are pluripotent stem cells that can be derived from kinds of sources such as bone marrow, umbilical cord, adipose tissue and dental pulp [79]. Akyurekli et al. reported that the therapeutic efficacy of MSCs appears to their paracrine factors [80]. Exosomes are now recognized as crucial paracrine mediators of MSCs, facilitating intercellular communication and preserving a dynamic, homeostatic microenvironment that supports tissue regeneration [81, 82]. Compared to MSCs, MSCs-Exo are easier to collect and store, and they face fewer ethical constraints [83]. MSCs-Exo, as a cell derivative, may play a crucial role in recovery after nerve injury [84].

Several researches have reported on the use of MSCs-Exo in the repairment of SNI in recent years [85–89]. Local injections of LPS pre-MSCs-Exo at nerve injury sites were found to deactivate the NF-κB/NLRP3 signaling axis by transferring TSG-6, promoting macrophage polarization to the M2 phenotype, thereby encouraging axon and myelin regeneration and enhancing nerve function recovery [85]. Zhang et al. found that MSCs-Exo carrying miR-181c-5p can reduce neuropathic pain induced by chronic compression of SCI by inhibiting neurogenic inflammation [86]. In a 12 mm sciatic nerve defect rat model, Zhang et al. found that human umbilical cord mesenchymal stem cells-derived exosomes (HUCMSCs-Exo) enhance the survival and migratory capacity of olfactory ensheathing cells under hypoxic conditions via activation of the BDNF signaling pathway, and the combined use of olfactory sensory cells and HUCMSCs-Exo can aid in restoring motor and sensory function after SNI [87]. Additionally, exosomes derived from bone marrow mesenchymal stem cells (BMSCs-Exo) can facilitate sciatic nerve regeneration, potentially through miRNA regulation of regenerative genes like VEGFA and S100b [88]. Moreover, studies on exosomes secreted by adipose-derived mesenchymal stem cells (ADMSCs-Exo) after acute sciatic neurotomy have shown that, compared to the control group, the exosome transplantation group exhibited increased axonal regeneration, improved function, and a higher quantity of regenerated nerve fibers due to the presence of signaling proteins like glial cell-derived neurotrophic factor, fibroblast growth factor-1, brain-derived neurotrophic factor, insulin-like growth factor-1, and nerve growth factor, which promote nerve survival and axon growth [89].

MSCs-Exo could present both challenges and opportunities in advancing regenerative strategies. Thus, comprehensive research is essential to unravel the mechanisms of MSCs-Exo in treating SNI while also evaluating their efficacy and safety.

#### MDSCs-Exo

Muscle-derived stem cell exosomes (MDSCs-Exo) overexpressing miR-214 significantly promote SCs proliferation and migration, increase neurotrophic factor levels in dorsal root ganglion neurons, stimulate axonal growth, and support the regeneration of neural structures after peripheral nerve injury by inhibiting PTEN protein expression and activating the JAK2/STAT3 signaling pathway [90]. However, research on using MDSCs-Exo for SNI repair is limited, and further studies are required to evaluate the safety and efficacy of this treatment approach.

### Schwann cell-derived exosomes (SCs-Exo)

SCs are a type of glial cell found in the PNS, and they play a vital role in maintaining neural homeostasis and promoting regeneration following peripheral nerve injury [91]. SCs supply nutrients to aid in axon regeneration and are the primary cell type responsible for myelin formation along the axon [92]. SCs-Exo, a second-generation derivative containing crucial informational substances from SCs, have been shown to play a significant role in neurodegeneration, neurodevelopment, and neuroprotection [93, 94].

SCs-Exo have been found to significantly enhance axon regeneration after SNI in vitro and in vivo. They induce alterations in growth cone morphology by reducing the activity of the GTPase RhoA, which is associated with growth cone collapse and axon retraction [95]. In a rat model of sciatic nerve compression, Sun and colleagues found that the ischemic and hypoxic microenvironment following SNI significantly reduced miR-146a-5p levels in SCs-Exo, prompting the conversion of M2 macrophages to M1 macrophages and thereby impeding axon regeneration and functional recovery [96]. Chen and colleagues created an animal model for sciatic nerve transection and demonstrated that Schwann cell-like cells (SCLCs) derived from human amniotic mesenchymal stem cells facilitate sciatic nerve recovery via the exosome-induced SOX2/FN1 signaling pathway [97]. Experiments showed that nerve grafts containing SCLCs-derived exosomes (SCLCs-Exo) significantly enhance sciatic nerve function recovery, reduce gastrocnemius atrophy, and promote axonal growth and myelination. Furthermore, SCLCs-Exo can stimulate SCs to migrate, proliferate, form myelin, and secrete neurotrophic factors [98].

Therefore, these findings deepen our understanding of how SCs-Exo contribute to repairing SNI, offering promising application prospects and valuable insights for their clinical use. Nevertheless, further research is required to fully realize the clinical potential of SCs-Exo in treating SNI patients.

### Blood-derived exosomes repair SNI

In recent years, a few studies have documented the use of blood-derived exosomes to treat SNI, though their precise mechanisms remain unclear and are likely to be the focus of future research. The findings indicate that the elevated expression of miR-21 in blood-derived exosomes following sciatic nerve ligation may partially contribute to reducing neuropathic pain [99]. Besides, Platelet-rich plasma-derived exosomes (PRP-Exo), harvested from platelet-rich plasma, exhibit tissue healing abilities and have been noted for their efficacy in addressing injuries in both the central and peripheral nervous systems [100, 101]. In a rat model of SNI, using ultrasound to target and destroy microvesicles enhances the delivery of PRP-Exo to the injury site, significantly concentrating PRP-Exo in the damaged nerve and improving therapeutic outcomes [102].

## Mechanisms of exosomes repairing SNI

Exosomes are small extracellular vesicles that play a crucial role in intercellular communication and have been widely studied for their potential in regenerative medicine, including nerve regeneration. Here’s a summary of how various stem cell-derived exosomes contribute to sciatic nerve recovery through different mechanisms (Table 1).

**Table 1:** Mechanisms of exosomes to repair SNI

Researchers	Exosomes	Signaling pathways/cytokines	Effect
Yin et al. [78]	ADSCs-Exo	Kpna2 through miRNA-26b	(1) Promote myelin regeneration
Liu et al. [42]	ADSCs-Exo	Bcl-2 mRNA/ Bax mRNA	(1) Promote SCs proliferation
Li et al. [85]	LPS pre-MSCs-Exo	NF-κΒ/NLRP	(1) Promote macrophages toward M2 (2) Promote axons and myelin sheath regeneration
Zhang et al. [86]	MSCs-Exo	MiR-181c-5p	(1) Reduce neuropathic pain
Sun et al. [96]	SCs-Exo	MiR-146a-5p	(1) Promote axon regeneration
Zhang et al. [87]	HUCMSCs-Exo	BDNF	(1) Improve OECS (2) Promote recovery of motor and sensory function
Zhao et al. [88]	BMSCs-Exo	VEGFA and S100b	(1) Promote sciatic nerve regeneration
Zeng et al. [90]	MDSCs-Exo	PTEN and JAK2/STAT3	(1) Promote ability of SCs (2) Support neurotrophic factors expression (3) Extend axon length
Chen et al. [97]	SCLCs-Exo	SOX2/FN1	(1) Promote sciatic nerve recovery
Rao et al. [106]	GMSCs-Exo	-	(1) Enhance the quantity and caliber of nerve fiber (2) Promote the formation of myelin
Yang et al. [114]	ECH-BMSCs-Exo	MEK/ERK	(1) Enhance myelin axon regeneration
Hori et al. [99]	Blood-derived exosome	MiR-21	(1) Alleviate neuropathic pain

ADSCs-Exo, adipose stem cell-derived exosomes; LPS pre-MSCs-Exo, lipopolysaccharide-treated mesenchymal stem cell-derived exosomes; MSCs-Exo, Mesenchymal stem cell-derived exosomes; SCs-Exo, Schwann cell-derived exosomes; HUCMSCs-Exo, human umbilical cord mesenchymal stem cell-derived exosomes; OECS, olfactory ensheathing cells; BMSCs-Exo, bone marrow mesenchymal stem cell-derived exosomes; MDSCs-Exo, muscle-derived stem cell-derived exosomes; SCLCs-Exo, Schwann cell like cells-derived exosomes; GMSCs-Exo, gingival mesenchymal stem cell-derived exosomes; ECH-BMSCs-Exo, electricity conductive hydrogels loaded with bone marrow mesenchymal stem cell-derived exosomes

Adipose Stem Cell-derived Exosomes (ADSCs-Exo) facilitate sciatic nerve recovery by modulating key signaling pathways, including down-regulating Kpna2, up-regulating Bcl-2 mRNA (which promotes cell survival), and down-regulating Bax mRNA (which promotes apoptosis) [42, 78].

Muscle-derived stem cells exosomes (MDSCs-Exo) can target and inhibit PTEN protein expression, a known negative regulator of the PI3K/Akt pathway, and activate the JAK2/STAT3 signaling pathway, both of which are critical for cell survival and proliferation [90].

MSCs-Exo affects axonal and myelin regeneration through multiple signaling pathways. LPS pre-MSCs-Exo focus on the inactivation of the NF-κB/NLRP3 signaling axis by transferring TNF-stimulating gene 6 (TSG-6) [85]. This action helps reduce inflammation, which is a significant factor in nerve damage and recovery. Besides, HUCMSCs-Exo could activate the brain-derived neurotrophic factor (BDNF) signaling, and play a crucial role for nerve growth and survival [87]. Moreover, BMSCs-Exo promote both vascular and neuronal growth through regulating the expression of VEGFA and S100b [88].

SC-Exo stimulate nerve regeneration by SOX2/FN1 axis, up-regulating SOX10, OCT-6, EGR2, MBP and MPI [97].

**Fig. 2:**
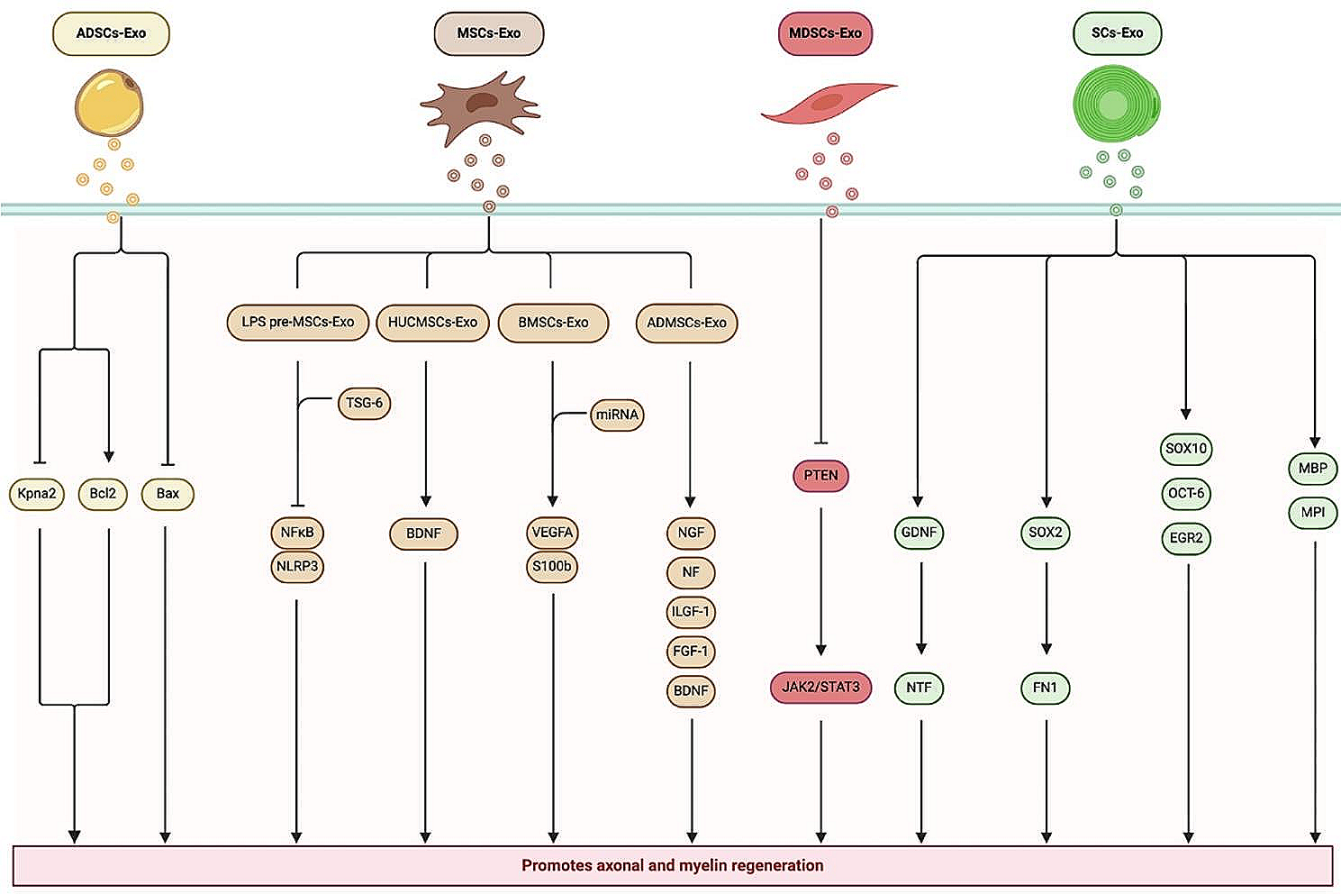
Mechanisms associated with exosome repair of SNI. ADSCs-Exo, Adipose stem cell-derived exosomes; MDSCs-Exo, Muscle-derived stem cell-derived exosomes; MSCs-Exo, Mesenchymal stem cell-derived exosomes; LPS pre-MSCs-Exo, Lipopolysaccharide-treated mesenchymal stem cell-derived exosomes; HUCMSCs-Exo, Human umbilical cord mesenchymal stem cell-derived exosomes; BMSCs-Exo, Bone marrow mesenchymal stem cell-derived exosomes; SCs-Exo, Schwann cells-derived exosomes

Overall, these exosomes harness various biological mechanisms to support nerve repair and regeneration, offering promising therapeutic avenues for conditions like SNI (Fig. 2).

## Exosomes combined with other methods to repair SNI

### Exosome combined with chitin catheter

Chitin catheters are ideal biomaterials for the construction of nerve catheters due to their good biodegradability, high antibacterial activity, high biocompatibility and non-toxicity [103]. Small-gap tubulization has considerable advantages over epineural sutures in terms of shortening the operative duration, enhancing the precision of axon docking and reducing the formation of neuromas [104]. However, hollow chitin catheters lack the biomechanical support and growth factors needed for nerve regeneration. On the contrary, exosomes can supply these critical factors, and when used in combination with chitin catheters, they enhance nerve regeneration by providing essential growth factors [105]. To increase the speed of nerve regeneration, it is critical to use a suitable transport medium. The chitin catheter effectively retains the bioactivity and bioavailability of exosomes while also demonstrating a strong affinity towards exosome binding.

Li et al. enhanced chitin catheters by incorporating polydopamine loaded with MSCs-Exo to bridge sciatic nerve defects measuring 2 mm in rats, and observed that the modified chitin catheter facilitated the sustained release of exosomes, which contributed to accelerated nerve healing and improved nerve function [105]. Rao et al. used a chitin catheter to transport exosomes and repair sciatic nerve defects in rats, discovering that the combined application of chitin catheters and gingival mesenchymal stem cell-derived exosomes (GMSCs-Exo) significantly enhanced nerve fiber quantity and caliber, promoted myelin formation, and improved nerve conduction and limb motor function [106]. Consequently, they advocate that the novel and effective integration of GMSCs-Exo with biodegradable chitin catheters represents a promising strategy for peripheral nerve repair [106]. Namini et al. facilitated axon regeneration and enhanced functional recovery of a sciatic nerve defect in rats by incorporating human endometrial stem cell-derived exosomes into the nuclear sheath of a fibroin/polylactic acid nerve catheter [84]. Yang et al. reported the therapeutic effect of a nerve guidance catheter (NGC) as an exosome carrier for the repair of SNI in rats. They initially encapsulated neurotrophin-3 into exosomes derived from ADSCs (ADSCs-Exo^NT-3^), which were then loaded into the NGC to create the ADSCs-Exo^NT-3^-NGC for bridging sciatic nerve defects in rats. After implantation of the NGC in vivo, ADSCs-Exo^NT-3^-NGC was found to significantly promote sural nerve regeneration and improve gastrocnemius function [107]. Generally, the chitin catheter, known for its good biocompatibility and degradability, offers an optimal biological microenvironment for the gradual release of exosomes, bridging nerve defects, and facilitating nerve regeneration.

### Exosomes carrying drugs

At present, there are no documented instances of using exosomes for drug delivery to the sciatic nerve. However, theoretically, it remains a viable method. Moreover, researchers have been modified exosomes to use them as therapeutic drug carriers due to their relatively small molecular structure, natural molecular transport properties, good biocompatibility, and ability to penetrate blood-spinal cord barrier [108, 109]. Zeng et al. [110], Gao et al. [111], and Yue et al. [112] demonstrated that exosome-based drugs, including azide-modified Ile-Lys-Val-Ala-Val peptide, berberine, and resveratrol, can effectively repair spinal cord injuries by reducing inflammation, promoting neuronal differentiation, and enhancing drug delivery across the blood-brain barrier. Exosomes, as drug carriers, offer numerous benefits. They enhance drug stability within the body, improve solubility, enable targeted delivery, and facilitate the transport of drugs across both the blood-brain and blood-spinal cord barriers. Consequently, exosomes represent a promising drug delivery system for PNI repair.

### Exosomes combined with hydrogels

**Fig. 3:**
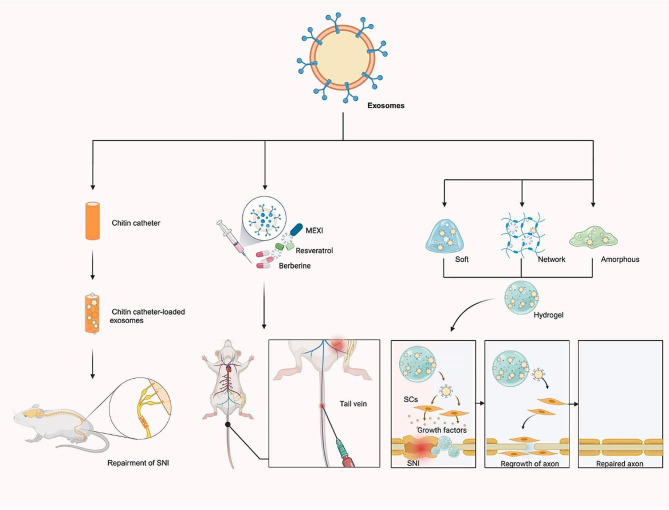
Exosomes combine with other strategies, mainly including chitin catheter, drugs and hydrogel, to repair SNI.SCs, Schwann cells

Hydrogel, known for its high-water content and diverse physical properties, is a type of polymer material. Recently, Liu et al. constructed a rat model of SNI and found that compared with hard hydrogels, soft hydrogels can better inhibit the inflammatory response caused by PNI through the rapid release of exosomes and promote nerve regeneration [113]. Furthermore, the study revealed that the release of exosomes, which is regulated by the hardness of the hydrogels, plays a crucial role in tissue repair, offering valuable insights for the clinical use of hydrogels [113]. In vitro studies by Yang et al. have shown that electricity conductive hydrogels (ECH) loaded with BMSCs-Exo can promote Schwann cell attachment and migration. Moreover, the exosomes in the system can regulate M2 macrophage polarization through the NF-κB pathway, thus mitigating inflammatory pain in diabetic PNI. Further, both in vitro and in vivo experiments showed that ECH loaded with BMSCs-Exo promotes myelin axon regeneration through the MEK/ERK signaling pathway, improving muscle denervation atrophy and aiding functional recovery [114]. Therefore, the combination of exosomes with hydrogel presents a promising strategy for the repairment of sciatic nerve injuries (Fig. 3).

## Challenges faced by exosome-mediated repair of SNI

### Low extraction yield

Due to the shortcomings of various methods for isolating exosomes, standardizing these techniques is crucial, yet there is currently no consensus. Significant differences in RNA and protein composition are observed across different isolation methods, highlighting the need for standardization of exosome composition for clinical applications [115]. Although ultracentrifugation is the predominant technique used in scientific research, exosomes isolated by this method tend to be less pure and may include other extracellular vesicles of similar diameter [116]. In addition, isolating exosomes from biological fluids or cell culture supernatants often results in a low yield [117–119].

### Low targeting

Optical imaging showed rapid accumulation of exosomes in the liver and spleen after intravenous injection on account of the presence of blood spinal cord barrier and blood brain barrier, indicating their unexpected biodistribution and short half-life, with exosomes having a much shorter half-life than nanocarriers (60 min versus several hours) [120]. Exosomes are also quickly eliminated by glomerular filtration, bile excretion and phagocytosis of the reticuloendothelial system [121], so the targeting of exosomes is very low. The incapacity to accurately characterize and quantify the relevant cargo carried by exosomes, as well as the incapacity to target them against specific receptors, leads to an off-target problem [40]. Low targeting and off-target lead to more rapid degradation of the drug and less effective treatment [122].

### Other challenges

**Fig. 4:**
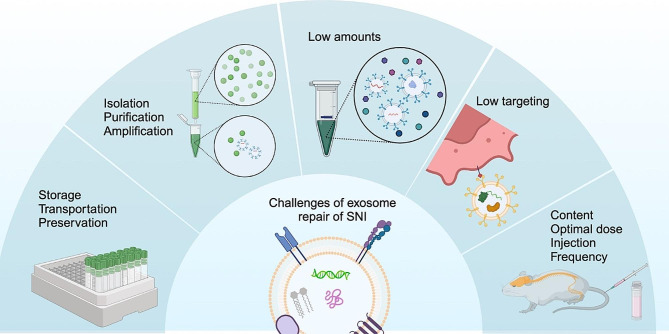
The dilemma facing exosome repair of SNI, such as low targeting, low mounts and hard storage

Exosomes face further challenges in the treatment of sciatic nerve damage. Firstly, the storage, transportation, and preservation of exosomes are critical. Secondly, various issues related to exosome sources and optimal culture conditions need to be resolved, including isolation, purification, replication, and the ideal dose, frequency, and method of administration of exosomes [83]. Third, the contents of exosomes require further study to identify which components have therapeutic potential for treating SNI and which may be harmful. Fourth, it is crucial to conduct more studies to explore the relationship between the frequency of injections, dosage, and the therapeutic efficacy of exosomes, and to determine whether single or multiple administrations could have adverse effects, all in order to sustain long-term efficacy [108] (Fig. 4).

## Conclusions

Currently, there is no definitive and effective treatment method that can fully restore nerve function after SNI. Exosomes sourced from various origins could aid in SNI repair by modulating multiple signaling pathways, including Kpna2, Bcl-2 mRNA/Bax mRNA, PTEN, JAK2/STAT3, TSG-6/NF-κΒ/NLRP3, BDNF, and VEGFA/S100, among others. Additionally, innovative approaches such as engineered exosomes, exosomes combined with chitin catheters, and exosomes paired with hydrogels show great promise in repairing SNI and are key focuses of ongoing research. However, there are substantial challenges in using exosomes for SNI repair, including low extraction yields, poor targeting accuracy, and challenges in achieving long-term drug delivery. The current research provides a robust theoretical basis for the preclinical exploration and practical application of exosomes in SNI treatment, offering significant potential for advancements. In summary, utilizing exosomes is proving to be an effective method for repairing SNI. In the future, comparing the effects of exosome treatment with other methods for SNI will be a new research direction.

## Data Availability

Not applicable.

## Abbreviations

SNI: Sciatic nerve injury
PNS: Peripheral nervous system
SCs: Schwann cells
ADSCs: Adipose-derived stem cells
MDSCs: Muscle-derived stem cells
MSCs: Mesenchymal stem cells
SCs-Exo: Schwan cell-derived exosomes
ADSCs-Exo: Adipose stem cell-derived exosomes
MDSCs-Exo: Muscle-derived stem cell-derived exosomes
LPS pre-MSCs-Exo: Lipopolysaccharide-treated mesenchymal stem cell-derived exosomes
HUCMSCs-Exo: Human umbilical cord mesenchymal stem cell-derived exosomes
BMSCs-Exo: Bone marrow mesenchymal stem cell-derived exosomes
SCLCs-Exo: Schwann cell like cells-derived exosomes
PRP-Exo: Platelet-rich plasma-derived exosomes
GMSCs-Exo: Gingival mesenchymal stem cell-derived exosomes
ADMSCs-Exo: Adipose-derived mesenchymal stem cells
ADSCs-Exo^NT−3^: Neurotrophin-3 into exosomes secreted from ADSCs
Gas6: Growth arrest-specific 6
NGC: Nerve guidance catheter
ECH: Electricity conductive hydrogels
TSG-6: TNF-stimulating gene 6
